# Conceptualisations of health in orthorexia nervosa: a mixed-methods study

**DOI:** 10.1007/s40519-022-01443-1

**Published:** 2022-07-21

**Authors:** Maddy Greville-harris, Catherine V. Talbot, Rachel L. Moseley, Laura Vuillier

**Affiliations:** Department of Psychology Faculty of Science and Technology, Bournemouth University, Poole, BH12 5BB UK

**Keywords:** Orthorexia nervosa, Disordered eating, Healthy eating, Dietary control, Health anxiety, Eating Disorders

## Abstract

**Purpose:**

Limited research has explored conceptualisations of health and healthy eating in orthorexia nervosa (ON). This mixed-methods study aimed to investigate how ‘health’ and ‘healthy eating’ are conceptualised by individuals at risk for ON. This study examined the potential relationships between health anxiety, beliefs about health controllability and orthorexic symptomatology in our broader sample.

**Methods:**

A total of 362 participants took a survey on health anxiety (measured with the HAQ), beliefs about the controllability of one’s own health (IMHLC) and ON symptomatology (E-DOS and EHQ). Participants ‘at risk’ for ON (*n* = 141), also completed an online qualitative survey about conceptualisations of health and healthy eating. Qualitative data were analysed using thematic analysis (part A). The PROCESS macro for SPSS was used for the quantitative analysis (part B).

**Results:**

Conceptualisations of health and healthy eating were complex. Four themes were generated from our qualitative data: ‘health is more than thin ideals’, ‘healthy food equals healthy mind’, ‘a body that works for you’ and ‘taking control of your body’. Our quantitative analysis revealed that health anxiety and beliefs in health controllability independently predicted ON symptomatology.

**Conclusion:**

A richer understanding of what health means in the context of ON is important, given the centrality of this concept to the proposed classification of this disordered eating style. Our findings highlight that both health anxiety and health controllability are important targets for future investigation, given their potential relevance to the aetiology and treatment of ON.

**Level of evidence:**

Level V, based on a descriptive study.

**Supplementary Information:**

The online version contains supplementary material available at 10.1007/s40519-022-01443-1.

## Introduction

Orthorexia nervosa (ON) is a disordered eating style, first described by Bratman as a pathological obsession with healthy eating [1]. While there is not yet a consensus on diagnostic criteria for ON, all published proposed criteria to date include the feature of obsessional preoccupation with 'healthy eating' that results in distress when personally created food rules are violated [2]. These food rules, as well as the definitions of 'healthy' and 'unhealthy', are subjective to each individual and may not conform to objective nutritional features or recommendations. Although a preoccupation with ‘eating healthily’ appears fundamental in ON, existing research has failed to address what ‘healthy eating’ really means to those with orthorexic symptoms, or how ‘health’ itself is construed by these individuals. As these concepts are embedded in proposed diagnostic criteria for ON, and fundamental to thinking around its aetiology and treatment, this research area warrants further attention.

Despite the pervasiveness in modern Western society of simplistic messages about food, eating, and their relationship to health, these messages are often contradictory and lacking in empirical support [3]. 'Healthy eating' is a complex construct that includes not just personal choice, but also social determinants of health, genetics, environment and culture [4]. Individual interpretations of 'healthy' and 'unhealthy' eating and foods are even more nuanced. Bisogni et al.’s comprehensive review of qualitative research [5] concluded that there was variation and complexity in terms of how people conceptualise eating ‘healthily’, with foods often being dichotomised as ‘good’ and ‘bad’ despite these categories not always matching those given by professionals. Eating ‘healthily’ was discussed not just in terms of particular food groups and nutrients, but also in terms of how foods were produced and prepared: ‘healthy foods’ were often defined as those which are ‘fresh’, ‘natural’ and ‘organic’ [6, 7], and a ‘healthy diet’ as one that was ‘balanced’ [8, 9]. Healthy eating was included as a contributing factor toward management or avoidance of illness or disease [10], as well as in enhancing psychosocial wellbeing [11].

When personal and societal conceptualisations of 'health' and 'healthy eating' are connected through the idea that health is personally controllable, they are referred to as 'healthism'. Healthism places the burden of responsibility for avoiding illness on each individual, ignoring the previously mentioned social determinants and other uncontrollable factors [12]. According to this doctrine, health is visibly manifested in bodies [13] and can be achieved through moral conduct and discipline [14]. This discourse implicates the individual’s own diet and behaviour as determining whether ‘good health’ is achieved and maintained.

While it is possible that healthism discourses play a role in the aetiology of orthorexic beliefs and behaviours, little research has explored whether individuals with ON believe that they have control over their own health. Some qualitative literature indicates a link between beliefs about health control and ON, suggesting that ON might be associated with a perceived need for control of health [15] as well as controllability of other aspects of participants’ lives [16]. In contrast, one quantitative study [17], examining locus of health control, found no statistical differences between participants in the ‘ON group’ compared with the ‘healthy eating’ group and the ‘normal’ eating group. However, this study was limited by their predominantly female college sample, and their categorising ‘healthy eating’ versus ‘ON’ groups based on a measure (the Eating Habits Questionnaire [18]) that does not provide such cut-offs.

‘Control’ is a topical concept in eating disorders (EDs), with a particular focus on maladaptive eating behaviours used as a means of controlling and changing one’s emotional experience, including alleviating anxiety [19]. Similarly, perhaps depending on the degree to which an individual implicitly or explicitly believes they control their health, it is possible that strict dietary behaviours in ON may reflect attempts to allay excessive anxiety and concerns around health. The presence and degree of health anxiety in ON are likewise under-researched, but one exploratory qualitative study [15] found that individuals who self-identified as having ON, reported health concerns as a trigger for their symptoms. Initial quantitative evidence also supports a relationship where increased anxiety around health may lead to increased preoccupation with strict health behaviours around eating [20, 21]. The first of these studies [20] was somewhat limited by the predominantly female university sample and the unsatisfactory psychometric properties of the ON measure that was implemented. In contrast, Barthel et al.’s study [21] employed an ON measure with much stronger psychometric properties, but only a small number of participants (*n* = 19) reported high levels of ON symptoms in their study. Nor did these studies consider potential interactions between these variables, such as might occur if health anxiety is associated with ON symptoms *only* when individuals perceive their health as controllable via lifestyle behaviours.

Given that preoccupation with ‘health’ and ‘healthy eating’ are proposed to be fundamental components of ON, it is important to explore how these terms are construed in the context of ON, and whether health anxiety and beliefs in health controllability are relevant factors to ON psychopathology. First, Part A of this study was devised to qualitatively explore conceptualisations of ‘health’ and ‘healthy eating’ in individuals scoring high on orthorexic symptomatology. Second, Part B of this study was devised to examine relationships between orthorexia symptoms and health anxiety and perceived controllability of health, exploring the main effects of both variables and moderation effects reflecting their interaction (Part B).

## Methods

### Participants

Participants were recruited via the Prolific participant recruitment website (a recruitment platform for online research). Only individuals aged 18 + , resident in the UK, and fluent English speakers were eligible to participate. While this study did not restrict study eligibility on the basis of diet or disordered eating, the study advert stated that researchers were looking for ‘clean eaters’ and people who identify with the label ‘orthorexia nervosa’ (or who currently feel preoccupied or overly focused on eating healthy or clean foods) to take part. Having excluded 24 incomplete datasets, 362 participants were included in the quantitative section of this study. The qualitative section comprised a subset of 141 participants who, scoring 25 $\ge$ on the English version of the Düsseldorf Orthorexia Scale (E-DOS [22]), were ‘at risk’ of ON. Sample demographics and descriptive data for the study variables are reported in Table 1.

**Table 1:** Demographic information and questionnaire descriptive data for the sample and the qualitative subsample

Demographic	Quantitative sample (*n *= 362)	Qualitative subsample (*n* = 141)
Gender		
Male	152 (42.0)	44 (31.2)
Female	204 (56.4)	94 (66.7)
Non-binary	6 (1.7)	3 (2.1)
Ethnicity		
White, White British or European	292 (80.7)	118 (83.7)
Black, African, Caribbean, or Black British	17 (4.7)	8 (5.7)
Asian or Asian British	43 (11.9)	12 (8.5)
Other Ethnic Group	10 (2.8)	3 (2.1)
Mental Health and Reported ED Diagnoses		
Reported self-identifying with the ON label	154 (42.5)	100 (70.9)
Reported a current ED diagnosis	14 (3.9)	13 (9.2)
Reported a past ED diagnosis	43 (11.9)	30 (21.3)
Reported that they believed they might currently have an undiagnosed ED	95 (26.2)	56 (39.7)
% Scoring 25 + (‘at risk’ for ON) on E-DOS	142 (39.2)	141 (100)
% Scoring 30 + (‘presence’ of ON) on E-DOS	77 (21.3)	76 (53.9)
Age	27.79 (8.89)	28.51 (8.22)
Body mass index	24.73 (5.26)	24.82 (5.34)
HAQ	1.14 (.61)	1.42 (.62)
E-DOS	22.33 (7.66)	30.28 (4.15)
MHLC (internal subscale)	25.22 (4.60)	25.72 (4.83)

Statistics reported are Frequency Count (Percentage) for gender, ethnicity, mental health diagnosis and E-DOS % cut-offs, and Mean (Standard Deviation) for the other variables

## Materials and procedure

This study was approved by the Bournemouth University Ethics committee and hosted on Qualtrics. After consenting to take part, participants completed a number of demographic questions (e.g., age, ethnicity and BMI) and a battery of standardised tests.

### The Health Anxiety Questionnaire (HAQ) [23]

The HAQ is a 21-item scale with good internal consistency [23]. Using a 4-point Likert scale from ‘0-not at all or rarely’ to ‘3-most of the time’, higher scores are indicative of greater health anxiety. Although there are no studies to date that have used this scale in the context of ON, the HAQ was developed based on the cognitive model of anxiety, has been found to have good psychometric properties [24], and has been used in a range of studies as an established measure of health anxiety (e.g. [24–26]).

### The Multidimensional Health Locus of Control Scale (Form A): Internality Subscale (IMHLC) [27]

To measure the extent to which participants believed that their health was within their control and determined by their own actions, the subscale of the Multidimensional Scale indicative of internal health locus of control (IMHLC) was used. It comprises six items scored using a 6-point Likert scale from ‘1- strongly disagree’ to ‘6-strongly agree’, with higher scores indicative of stronger beliefs that health is controllable by one’s own actions. The IMHLC is a widely used study of health locus of control, with one other study to date implementing this measure in the context of ON research [17]

### Düsseldorf Orthorexia Scale, English version (E-DOS) [22]

A measure of orthorexic behaviour with good psychometric properties [22], the E-DOS requires participants to rate ten items on a 4-point Likert scale from ‘1- this does not apply to me’ to ‘4-this applies to me’. The original German version of the scale showed high test–retest reliability and high internal consistency [28]. This scale was translated into English to create the E-DOS via the back translation process [22]. The English version of the scale has shown good internal consistency (Cronbach’s alpha = 0.84) [22]. In line with Chard’s study [22], scores on the E-DOS of 30 $\ge$ are used to indicate ‘presence’ of ON, with scores between 25 and 29 (95th percentiles) indicating ‘at risk’ of ON. In our study, a subset of participants was identified as ‘at risk’ of ON (scoring 25 $\ge$) and redirected to complete the qualitative part of the study.

### The Eating Habits Questionnaire (EHQ) [18]

As there is no consensus around the optimal measurement of ON [29, 30], the 21-item EHQ was included solely as a confirmatory measure to corroborate our quantitative analyses. Items assessing cognitions, behaviours and emotions related to an extreme preoccupation with healthy eating are rated on a 4-point Likert scale from ‘1-false, not at all true', to ‘4- very true’, with higher scores indicative of likely difficulties in this area. The EHQ is increasingly used as a promising measure of ON across a range of studies [30, 31].

### Qualitative survey

In addition, participants (n = 141) who were indicated, by their E-DOS scores (25 ≥), as being ‘at risk’ of ON were asked eight open-ended questions (see Supplementary Materials 1) which explored their ideas of health and healthy eating. While eligibility for this part was based on the less stringent E-DOS cut-off, 53.9% of participants fell above the more severe cut-off (30 ≥) reflecting ‘presence of ON’.

## Data analysis

For Part A, thematic analysis was used to analyse the qualitative survey responses of participants ‘at risk’ of ON. Data were imported into NVivo Pro 12.5 (QSR International) for initial coding. Braun and Clarke’s stages of analysis [32] were then followed including: familiarisation with the data, data coding, generation of initial themes, developing and reviewing themes, defining and naming themes, and writing up the analysis. Two researchers (CT and MGH), both with extensive experience in qualitative methods in ED research, carried out the analysis and the final themes were refined through collaboration and discussion.

For part B, all participants were included. The PROCESS macro for SPSS (version 28) model 1 was used to examine the relationship between health anxiety (HAQ scores), beliefs of health controllability (IMHLC scores) and ON symptoms (E-DOS scores). Modelling the IMHLC variable as a moderator allowed us to scrutinise its direct relationship to ON symptomatology and any interaction effects it exerted on the association between health anxiety and ON symptoms. Bootstrapping sampling was set to 5000 and all variables were mean-centred. This analysis was corroborated by the Eating Habits Questionnaire (EHQ) [18] modelled as the outcome (See Supplementary material 2).

## Results

### Part A: conceptualisations of health- qualitative analysis

As outlined in Table 2, four themes were generated from the data.

**Table 2:** Themes and subthemes

Theme	Subthemes
(T1) Health is more than thin ideals	
(T2) Healthy food equals healthy mind	
(T3) A body that works for you	Food as functional
	Unhealthy food is dangerous
(T4) Taking full control of your body	Feeling in control of my eating and in control of my life
	Dealing with transgressions

#### Theme 1: health is more than thin ideals

While for many participants the concept of health was intertwined with ideas about weight ideals and low-calorie foods, a ‘healthy body’ went beyond thin ideals, with many describing a body that was not only low in body fat but also toned with visible muscle definition.

“Healthy means that I am at a weight and shape that I like the look of, flat stomach and toned, no fat” (*P56, male, 40 years, E-DOS score: 31*)

Through pursuing this body ideal, respondents explained that they would feel more confident in their body and less worried about judgment from others.

“I will consider myself... healthy when I no longer worry about what people think about my body...I won’t feel healthy if I went to a beach and everyone could see that I wasn’t in great shape” (*P54, male, 25 years, E-DOS score: 26*)

In contrast, ‘fat’ bodies were frequently positioned as ‘unhealthy bodies’, associated with laziness, lack of control and sickness. Interestingly, one participant differentiated between ‘skinny fat’ and ‘fat’, highlighting that health went beyond the aesthetic, also including the nutritional value of certain foods.

“…there's two ways of looking unhealthy you can be skinny fat or fat. skinny fat is where someone is slim but has a good metabolism so eats junk food and dose no form of exercise this person would look skinny but would have a high risk of heart disease. Someone who is 'fat' … has very similar habits but eats much more and has a slower metabolism.” (*P71, female, 24 years, E-DOS score: 28*)

#### Theme 2: Healthy food equals healthy mind

‘Health’ was not just associated with a certain ‘look’ but also a certain ‘feeling’. Living a healthy lifestyle was described in terms of the consequences for positive mental health and wellbeing, with greater confidence, energy and positive outlook mentioned as important motivators for a healthy diet.

“…I found the better I ate, the better I felt, I don't feel lethargic or depressed, I know that I'm putting the right things in my body and in turn it's helping me to feel better physically and mentally” *(P100, female, 21 years, E-DOS score: 32)*

In contrast, eating ‘bad foods’ were seen to contribute to low mood, low self-esteem, low motivation, as well as feelings of guilt and anxiety.

“Being unhealthy to me mainly means having a sedentary lifestyle and mostly eating junk food. Its much more than just food and exercise, but a lack of quality in these things can make you feel bad within yourself in terms of mood and motivation.” *(P123, female, 26 years, E-DOS score: 27)*

#### Theme 3: a body that works for you

Across two subthemes, many participants discussed the role of healthy eating in enhancing performance and reducing susceptibility to illness.

##### Food as functional

Participants emphasised the importance of having a body that worked at optimal performance, with many feeling personally responsible for achieving this goal.

“I want to perform at my best. My diet should support me to achieve my goals.” *(P19, male, 25 years, E-DOS score: 31)*

Food was discussed as ‘fuel’ and participants recognised the importance of getting the ‘right’ nutrients to operate at peak performance. The focus was on food that was ‘efficient’, maximised energy output and nourished the body.

“… food is for fuelling your body and maximising the energy output for the food eaten. Other people eat empty calories and fatty foods. they are doing it all wrong” *(P2, 36 years, female, current diagnosis of Other Specified Feeding and Eating Disorder- OSFED, E-DOS score: 38)*

##### Unhealthy food is dangerous

‘Unhealthy’ food (or elements such as chemicals, toxins and sugars) were sometimes described as pathogenic, linked to inflammation, heart disease and even cancer. Participants associated illness with eating the ‘wrong’ foods, such that many felt responsible for staying well and avoiding ‘preventable’ illness.

“… our bodies need certain vitamins to keep it healthy if we don't have them we give cancer a chance to grow so we must stay away from processed food which grows cancer” *(P124, female, 43 years, E-DOS score: 36)*

One participant indicated that these concerns were exacerbated by the COVID-19 pandemic.

“Being healthy means putting only the purest, cleanest food in my body.if i want my body to survive, specially during a pandemic it needs to be clean food....” *(P2, female, 36 years, E-DOS score: 38)*

Consequently, participants followed various different food rules, including controlling food type and composition.

“Eating high quality food. Organic, free range, hormone free, grass fed etc.…. I'm not trying to lose weight. I'm trying to put clean, healthy, pesticide-free, hormone-free food in my body” *(P108, female, 51 years, E-DOS score: 31).*

#### Theme 4: taking full control of your body

Across two subthemes, participants described eating healthily as a way to control their bodies. Taking control and distress at dietary transgressions were discussed.

##### Feeling in control of my eating and in control of my life

Participants reported that ‘healthy eating’ provided a way of taking control of their body, and in some cases, gave a sense of control over their life.

“Being in control of what I eat and not the other way around - most everyone is controlled by food … and this is the cause of disease.” *(P141, non-binary, 30 years, E-DOS score: 35*)

In contrast, ‘unhealthy’ people were construed as not controlling their food intake or resisting urges, instead eating impulsively and mindlessly.

“Unhealthy …is giving in and eating what the body impulsively tells you it wants. … Being unhealthy is being weak to food desires.” *(P110, Female, 18 years, E-DOS score: 38)*

Pursuit of a healthy diet often involved controlling calories, tracking intake, and avoiding whole food groups.

“I have calculated my macros and calorie allowance for the day, I scan everything in prior so I know what I can eat ...” *(P84, female, 26 years, E-DOS score: 32)*

##### Dealing with transgressions

Many participants described distressing emotions (guilt, anger, anxiety, depression) or physical sensations (feeling unwell) in response to lapses in their ‘healthy’ diet, often engaging in self-criticism and self-judgment in response to these dietary transgressions.

“[I would] freak out, likely to be disgusted with myself and feel strong anxiety about the effects” *(P50, male. 29 years, E-DOS score:38)*

One dietary lapse was often discussed in terms of catastrophic consequences, such as being a sign of failure, leading to certain weight gain or reverting to old habits.

“It feels like if I ate food outside of this diet, i would most likely lose control of my eating habits, I would spiral and put on weight” *(P100, female, 21 years, E-DOS score:32)*

Some participants preferred to miss meals rather than eat ‘unhealthy’ foods, with many discussing compensatory behaviours following a dietary transgression, including restriction or increase in healthy eating/exercise for the next day.

“I would starve myself later on or go on a run and feel very guilty” *(P84, female, 26 years, E-DOS score: 32)*

### Part B: Health anxiety, health controllability and ON symptomatology—Quantitative Analysis

In this study, higher levels of health anxiety (*b* = 5.89, *p* < *0.0*01) and health controllability (*b* = 0.22, *p* = 0.005) were indeed associated with greater ON symptomatology (R^2^ = 0.24, *F* (3, 358) = 38.48, *p* < *0.0*01). The absence of any significant moderation effect (*p* = 0.2444), suggested that the highly significant relationship between health anxiety and ON symptomatology did not differ as a function of health beliefs. These findings of two independent main effects remained consistent when EHQ scores were modelled as an alternative index of ON symptoms (see Supplementary materials, 2).

## Discussion

Although extant literature considers conceptualisations of ‘health’ within the general population [5], little is known about how individuals with ON symptomatology conceptualise ‘healthy eating’ and a state of health, *or* about the relevance of health-related emotions and cognitions to ON symptomatology. Given the prominence of ‘healthy eating’ as a construct in proposed diagnostic criteria for ON, the present mixed-methods study aimed to address these gaps.

In the qualitative part of our study, ‘health’ emerged as a complex term which albeit intertwined with ideas about weight and weight management, incorporated the ‘fit’ body ideal with visible body definition and tone. While health was associated with physical functionality and fitness, it also referred to mental health, with a ‘healthy’ diet beneficial for confidence and wellbeing. For some participants, orthorexia resembled an extreme manifestation of healthism in which adherence to a ‘healthy’ diet established proof of character, and dietary ‘transgressions’ resulted in feelings of failure, worthlessness, anxiety, or guilt. While similar feelings are described in other qualitative studies of ON [33], they are also frequently described in the broader literature around ED [34].

Though there are obvious benefits of adhering to healthy diets, there are inherent problems with healthism discourses which construe physical and mental health and healthy eating as the responsibility of the individual [35]. Aside from reinforcing stigma around different bodies [36], achieving health becomes a continuous effort of managing risk and health ‘threats’ through personal investment and commitment [37]. In the context of orthorexia and disordered eating, healthism legitimises the message that health requires constant vigilance and self-restraint. The idealisation of fitness as well as thinness makes body ideals even more unattainable [38], with both ‘fitspiration’ and ‘thinspiration’ posts associated with guilt about weight and shape, restrictive eating [39], decreased body satisfaction [40] and less positive mood [41]. These attitudes in our participants, and the impact and compensatory aftermath of perceived dietary transgressions, are deeply concerning, particularly as most of the qualitative subset (90.8%) did not report a current diagnosis of an eating disorder and reported a body mass index in the ‘healthy’ range; furthermore, nearly half fell within the ‘less severe’ bracket of E-DOS scores (25–30) indicative of ‘conspicuous eating behaviour’ rather than ‘presence’ of ON. In addition, over a third of the qualitative sample (39.7%) reported that they thought they might have an undiagnosed eating disorder, suggesting that their potential difficulties with eating were currently not being clinically recognised or addressed.

Themes interpreted in the qualitative analysis were echoed in our quantitative analysis. Participants discussed anxiety-provoking beliefs that the ‘wrong’ diet would have a pathogenic influence on body and health, using strict dietary behaviours and exercise to control body and performance. Similarly, our quantitative analysis indicated the distinct importance of both health anxiety and beliefs in health controllability for ON symptomatology. The relationship with health anxiety corroborates previous quantitative findings in large samples involving few, if any, participants with ON [20, 21] and extends these findings by showing this association to operate regardless of the extent to which participants believed they could influence their health. While higher scores in health anxiety were associated with greater ON symptoms at all levels of belief in health controllability, greater belief in health controllability was instead itself an independent predictor of ON symptoms. This finding contradicts previous statistical comparisons which failed to highlight increased belief in health controllability in people with potential ON [17]. The previous study may have been underpowered and insufficiently stringent in group categorisation, but also tested group differences across multiple locus of control subscales to compare scores for ‘healthy eating’, ‘normal eating’ and ‘ON symptom’ groups. As such, these researchers were using different methodology and different analyses which may also explain the contradictory findings. In contrast, the qualitative and quantitative data in our study highlight both health anxiety and health controllability as important targets for future investigation, given their potential relevance to the aetiology and treatment of ON.

### Strengths and limitations

This study is the first focusing on conceptualisations of health in the context of ON, and benefitted from a mixed-method approach which afforded both examinations of factors associated with ON symptomatology and richer exploration of how individuals conceptualise ideas of ‘health’ and ‘healthy eating’. This research was also able to address some limitations of previous research in our recruitment of a large, more male/female-balanced sample and our use of two validated measures of ON. In targeting individuals who self-identified with the label of ON during recruitment, this study also gathered qualitative data from a large sample of individuals who displayed high levels of ON tendencies. However, the study is also subject to several limitations. First, although our survey allowed us to gather broad data from a large sample, more focused qualitative work using semi-structured interviews is important for in-depth exploration of conceptualisations of health. Relatedly, unfortunately, reliance on self-report is necessitated while ON is not included in diagnostic manuals. While self-report information about eating disorder diagnoses was gathered for this research, the researchers did not formally confirm these, or whether ON developed in the recovery phase of their eating disorder (see [42]). Finally, as cross-sectional data forbids interpretations of directionality in relationships, longitudinal research is required to corroborate the relevance of health anxiety and health controllability beliefs to ON.

## Conclusion

Our qualitative and quantitative data suggest that beliefs and emotions around health and healthy eating are indeed fundamental to ON. Participants high in orthorexic tendencies discussed anxiety-provoking beliefs about the pathogenic influence of unhealthy foods, using strict dietary behaviours and exercise to control their body and health. Participants also described concerning compensatory behaviours following transgressions from their ‘healthy’ diet. While greater health anxiety and stronger beliefs in health controllability both predicted ON symptomatology, future research must examine directionality in these relationships to ascertain the importance of these variables for the aetiology and treatment of ON.

### What is already known

While ‘preoccupation with healthy eating’ is believed to be central to ON, no research has explored how healthy eating and health are understood by people with ON. In addition, limited research has explored relationships between health anxiety, health controllability and ON.

### What this study adds

Health is complexly construed by people with ON, intertwined with the idealisation of thinness and fitness, with moral judgements of character, and with feelings of responsibility to avoid illness. Healthy eating is conceptualised as a means of controlling one’s life and health. Health anxiety and beliefs in health controllability were both associated with ON symptomatology implicating both of these factors as important in the aetiology and potential treatment of ON.

## Supplementary Information

Below is the link to the electronic supplementary material.

Supplementary file1 (DOCX 15 KB)

## Data Availability

Data are available on request from the authors.
