# Supplementary material for: Conceptualisations of health in orthorexia nervosa: a mixed-methods study
Source: Eat Weight Disord. 2022 Jul 21;27(8):3135–43. doi: 10.1007/s40519-022-01443-1 (PMC9301897; doi:10.1007/s40519-022-01443-1)
Supplement: Supplementary file 1 — Supplementary file1 (DOCX 15 KB) [file 40519_2022_1443_MOESM1_ESM.docx]

**Conceptualisations of Health in Orthorexia Nervosa: A Mixed Methods Study**

Maddy GREVILLE-HARRIS, Catherine V. TALBOT, Rachel L. MOSELEY, and Laura VUILLIER

Department of Psychology Faculty of Science and Technology, Bournemouth University, Poole, UK, BH12 5BB.

^*^Corresponding author: Maddy Greville-Harris.

Email: mgrevilleharris@bournemouth.ac.uk

**Supplementary Materials 1- Qualitative Survey Questions**

In this section we would like to ask you some more detail about your experiences and views about healthy eating and your diet. Please provide as much detail as possible when answering the following questions:

- What does ‘being healthy’ mean and look like to you? (Feel free to give examples if it helps you to explain.)
- What does ‘being unhealthy’ mean and look like to you? (Again, feel free to give examples if it helps you to explain.)
- What foods count as ‘healthy’ foods? Why do you consider these foods to be healthy?
- What foods count as ‘unhealthy’ foods? Why do you consider these foods to be unhealthy?
- Was there a time in the past when your idea of ‘healthy eating’ looked different to how it does now? How did it look in comparison?
- What are your motivations for eating your chosen diet?
- How might you think, feel and act if you were in a situation where you were unable to follow your chosen diet?
- How do you decide what food is good for you? Please describe your thought process when deciding what to eat.

**Supplementary Material 2- Additional Moderation Analysis using Eating Habits Questionnaire as the Measure of Orthorexic Tendencies.**

To corroborate our primary moderation analysis with an additional measure of ON symptoms, we substituted E-DOS scores for scores on the Eating Habits Questionnaire (EHQ). As in our primary analysis, there was a significant association between health anxiety and EHQ scores (*b* = 9.55, *p* < .001; *R2* = .27, *F*(3, 358) = 43.20, *p* < .001), and a main effect of having higher beliefs in health controllability (*b* = .61,*p* < .001). There was no significant moderating effect of health controllability on the association between health anxiety and EHQ scores.
